# Pain management policies and practices in pediatric emergency care: a nationwide survey of Italian hospitals

**DOI:** 10.1186/1471-2431-13-139

**Published:** 2013-09-10

**Authors:** Pierpaolo Ferrante, Marina Cuttini, Tiziana Zangardi, Caterina Tomasello, Gianni Messi, Nicola Pirozzi, Valentina Losacco, Simone Piga, Franca Benini

**Affiliations:** 1 Unit of Epidemiology, Bambino Gesù Children’s Hospital, Viale Ferdinando Baldelli 41, Rome 00146, Italy; 2 Department of Emergency Medicine, University Hospital, Padova, Italy; 3 Department of Emergency Medicine, Bambino Gesù Children’s Hospital, Rome, Italy; 4 Department of Emergency Medicine, Burlo Garofolo Children’s Hospital, Trieste, Italy; 5 Pediatric Department, University Hospital, Padova, Italy

**Keywords:** Pediatric pain management, Algometric scales, Emergency care, Policies

## Abstract

**Background:**

Pain experienced by children in emergency departments (EDs) is often poorly assessed and treated. Although local protocols and strategies are important to ensure appropriate staff behaviours, few studies have focussed on pain management policies at hospital or department level. This study aimed at describing the policies and reported practices of pain assessment and treatment in a national sample of Italian pediatric EDs, and identifying the assocoated structural and organisational factors.

**Methods:**

A structured questionnaire was mailed to all the 14 Italian pediatric and maternal and child hospitals and to 5 general hospitals with separate pediatric emergency room. There were no refusals. Information collected included the frequency and mode of pain assessment, presence of written pain management protocols, use of local anaesthetic (EMLA cream) before venipuncture, and role of parents. General data on the hospital and ED were also recorded. Multiple Correspondence Analysis was used to explore the multivariable associations between the characteristics of hospitals and EDs and their pain management policies and practices.

**Results:**

Routine pain assessment both at triage and in the emergency room was carried out only by 26% of surveyed EDs. About one third did not use algometric scales, and almost half (47.4%) did not have local protocols for pain treatment. Only 3 routinely reassessed pain after treatment, and only 2 used EMLA. All EDs allowed parents’ presence and most (17, 89.9%) allowed them to stay when painful procedures were carried out. Eleven hospitals (57.9%) allowed parents to hold their child during blood sampling. Pediatric and maternal and child hospitals, those located in the North of Italy, equipped with medico-surgical-traumatological ED and short stay observation, and providing full assessment triage over 24 hours were more likely to report appropriate policies for pain management both at triage and in ER. A nurses to admissions ratio ≥ median was associated with better pain management at triage.

**Conclusions:**

Despite availability of national and international guidelines, pediatric pain management is still sub-optimal in Italian emergency departments. Multifaceted strategies including development of local policies, staff educational programs, and parental involvement in pain assessment should be carried out and periodically reinforced.

## Background

Assessment and safe management of pain in children is a crucially important but challenging task for the emergency care team [1]. Frequently reported barriers include difficulties in measuring pain in pre-verbal patients, diagnostic uncertainty and fear that drugs may mask clinical signs, concerns about medication adverse effects and potential for addiction, lack of space, understaffing and time constraints [2-4].

Pain experienced by children in emergency departments (EDs) is often poorly assessed and treated [5]. Pediatric patients are reported to receive less analgesia than adults with similar diagnoses or surgical procedures, and to be prescribed inadequate dosages of analgesic at discharge [6,7]. Very young children and infants are less likely to receive pain assessment and treatment than school age children and adolescents with comparable pain levels [8,9]. Minor but painful procedures are commonly performed without pain management [10]. Pain reassessment after treatment is uncommon [7,11].

Although the importance of local protocols and targeted strategies in ensuring consistent and appropriate staff behaviour is recognized [12,13], few studies have focussed on the pain management policies and strategies at hospital or department level. In the late 90s a postal survey carried out in England showed that only 20% of EDs had a policy for pain management in children; 35% routinely assessed and recorded children’s pain scores, and 50% provided formal training for pain management to the staff [14]. At about the same time in the US Krauss et al. [15] found that EDs based in children’s hospitals performed significantly more sedations than those in general hospitals, matching for pediatric volume. More recently, Tourtier et al. [16] reported that a written protocol for managing pain in children was available in 65% of EDs responding to a postal questionnaire in the Île de France. A similar survey carried out in the Netherlands found that 35% of protocols for acute pain management did not addressed children; 73% required a diagnosis before pain relief; 6% did not include opioids, and 36% did not allow intravenous opioids [17]. More encouraging results were reported by the PREDICT (Paediatric Research in Emergency Departments International Collaborative) group in Australia and New Zealand, where 92% of pediatric EDs had pain management policies or guidelines, 92% taught pain management topics in education programmes, and 62% used mandatory pain competencies. However, only 15% had quality improvement programmes for pain reduction [18].

In Italy, a book on Pain in Children was published in 2010 by the Italian Ministry of Health, providing detailed theoretical and practical information about pain assessment and treatment according to age and type of pain [19]. However, it is not known to what degree these recommendations have been translated into local protocols and policies in Italian hospitals.

This paper is aimed at describing the policies and reported practices of pain assessment and management in a national sample of Italian pediatric EDs, and identifying the associated structural and organisational factors.

## Methods

All the Italian pediatric and maternal and child hospitals (n.14) were invited to participate in the study, together with 5 general hospitals with separate pediatric emergency room (ER). There were no refusals. Taken together, in 2010 the 19 ERs performed over 635000 annual pediatric admissions.

For the purposes of this study, the term emergency department (ED) was used to include triage, emergency room (ER), and short stay observation (SSO). Information about the EDs policies regarding pain assessment and treatment at triage and in the emergency room was collected by means of a structured questionnaire. The following aspects were investigated: frequency of pain assessment (never, sometimes, always), use of algometric scales, recording of pain assessment results in clinical records, presence of pain treatment protocols, use of local anaesthetic (EMLA cream) when venipuncture is anticipated, and role of parents in the ER. General information on the hospital and on the ED, including medical and nursing staffing in full time equivalents and annual number of pediatric admissions, were also collected. The questionnaire was piloted in three hospitals with different characteristics (pediatric, maternal and child, and teaching hospital). The final version was presented in a meeting with the representatives of the 19 EDs. Data collection took place in April-October 2010. All the 19 distributed questionnaires were completed (response rate 100%).

As the study did not involve any collection or analysis of personal data regarding human participants, but only hospitals and policies, according to Italian law requirements for informed consent and approval by Ethic Committee did not apply. Specifically, in Italy ethical review is mandatory only for clinical trials on pharmaceutical products (http://www.agenziafarmaco.gov.it/sites/default/files/Decreto_Legislativo_n._211_del_24_giugno_2003.pdf) and for observational studies on use of medicines by human participants (http://www.agenziafarmaco.gov.it/sites/default/files/det_20marzo2008.pdf).

Statistical analysis was carried out by the Epidemiology Unit of Bambino Gesù Children’s Hospital. Results were summarized as absolute and relative frequencies for categorical variables, and means and standard deviation (sd) for continuous variables. Proportions were compared using the Fisher’s exact test, and means by analysis of variance.

We used Multiple Correspondence Analysis (MCA) to explore the association between the characteristics of hospitals and EDs and their policies and practices regarding pain management in children.

MCA is a multivariable statistical technique suitable to detect and represent graphically the underlying structure (i.e. “dimensions”) in a given data set [20]. Variables related to the study outcomes (called “active” variables) are used to build the graph (i.e. “map”), where categories with similar patterns are represented by points clustered together in space. As a second step, additional variables related to the characteristics of the sample (so called “passive” variables, or predictors) may be plotted over the map to explore their relationship with the underlying dimensions identified by the active variables. MCA requires categorical type of variables, and is particularly useful when large number of variables are involved [20]. Binomial coding (yes/no) is advisable for sake of clarity.

The following variables were used to build the map (“active” variables):

I. *pain management at triage:* pain assessment (coded as always versus sometimes/never); assessment results recorded in clinical records, use of algometric scales, pain as sufficient criterion for priority coding, availability of nursing protocols for pharmacological and non-pharmacological pain treatment (all coded as yes or no); and use of EMLA cream when venipuncture is anticipated (always to sometimes versus never/almost never).

II. *pain management in the ER:* pain assessment (always versus sometimes/never); results recorded in clinical records, use of algometric scales, written protocols for pain treatment in ER (all coded as yes or no); pain reassessment after treatment (always/often versus sometimes/never); and possibility for parents to hold their child during blood sampling (yes or no).

Variables plotted on the map as “passive” (i.e. predictors) included: geographical area (North, Centre, or South of Italy); hospital type (pediatric or maternal and child versus general); ED type (medico-surgical-traumatological –MST- versus otherwise); availability of short stay observation, SSO (yes or no); availability of pediatric triage (24 h or <24 h); triage method (full assessment versus first look only); and staffing, measured as number of full time equivalent nurses and physicians per ED annual pediatric admissions (coded as ≥ or < median value).

Two main dimensions, represented by orthogonal axes, explained 82% of the variance in the active variables (see “Results”). According to Greenacre and Pardo [21], a five degrees anticlockwise rotation of the axes was performed to improve the lining up of the two sets of points to the axes in the map.

Statistical analysis was carried out with the Stata package (StataCorp. 2009. Stata Statistical Software: Release 11.0. College Station, TX: StataCorp.).

## Results

The characteristics of the participating hospitals and emergency departments by geographical area are presented in Table 1. Fourteen were academic hospitals, and 4 belonged to the network of clinical research institutes (Istituto di Ricovero e Cura a Carattere Scientifico, IRCCS) of the Italian Ministry of Health. Fifteen hospitals had a MST emergency department, and offered SSO. Full assessment pediatric triage was available on a 24 h basis in all hospitals but two. The overall mean number of annual pediatric admissions was 33400 (range 11000 to 107000). The lowest annual frequencies belonged to the 5 general hospitals (11000 to 30000, data not shown in table).

**Table 1:** Characteristics of participating hospitals by geographical area

	**North**	**Centre**	**South**	**Total**
	**n (%)**	**n (%)**	**n (%)**	**n (%)**
**Type of hospital:**				
General	3 (27.3)	2 (40.0)	0 (0.0)	5 (26.3)
Pediatric/maternal and child	8 (72.7)	3 (60.0)	3 (100.0)	14 (73.7)
**Academic hospital:**				
No	3 (27.3)	0 (0.0)	2 (66.7)	5 (26.3)
Yes	8 (72.7)	5 (100.0)	1 (33.3)	14 (73.7)
**Clinical Research Hospital (IRCCS):**				
No	8 (72.7)	4 (80.0)	3 (100.0)	15 (78.9)
Yes	3 (27.3)	1 (20.0)	0 (0.0)	4 (21.0)
**Type of pediatric emergency department:**				
Medical only	1 (9.1)	0 (0.0)	1 (33.3)	2 (10.5)
Medico-surgical or medico- traumatological	2 (18.2)	0 (0.0)	0 (0.0)	2 (10.5)
Medico-surgical-traumatological (MST)	8 (72.7)	5 (100.0)	2 (66.7)	15 (78.9)
**Short Stay Observation (SSO):**				
No	1 (9.1)	2 (40.0)	1 (33.3)	4 (21.0)
Yes	10 (90.9)	3 (60.0)	2 (66.7)	15 (78.9)
**Triage carried out:**				
<24 h/day	0 (0.0)	0 (0.0)	2 (66.7)	2 (10.5)
24 h/day	11 (100.0)	5 (100.0)	1 (33.3)	17 (89.5)
**Triage method:**				
First look only	1 (9.1)	1 (20.0)	1 (33.3)	3 (15.8)
Full assessment	10 (90.9)	4 (80.0)	2 (66.7)	16 (84.2)
**Number of annual pediatric admissions (x 1000):**				
Mean (sd)	26.7 (10.9)	33.3 (15.9)	58.4 (43.6)	33.4 (21.6)
**Physicians to admissions ratio (PR) (x 10000):**				
Mean (sd)	3.7 (1.9)	3.5 (1.6)	2.7 (1.4)	3.5 (1.7)
**Nurses to admissions ratio (NR) (x 10000):**				
Mean (sd)	6.0 (1.9)	7.2 (1.2)	4 (0.3)	6 (1.8)

Compared to those in the North and Centre, the three hospitals in the South of Italy were less likely to be academic hospitals, and to have full assessment pediatric triage available over the 24 hours. They also had the largest mean number of annual pediatric admissions (p = 0.07), and lower staff to admissions ratios, particularly for nurses (p = 0.06).

Table 2 shows the policies towards pain management at triage and in the ER. Only 9 hospitals reported to routinely (i.e. “always”) assess children pain at triage, and only 8 in the ER. Thirteen hospitals (68.4%) used algometric scales at triage, and 9 (47.4%) in the ER. The visual analog (VAS) and the Wong-Baker faces pain scales were the most frequently used instruments (44.4% and 38.9% of hospitals respectively, data not shown in table). Recording of results of pain assessment in clinical records was reported at triage by 14 (73.7%) hospitals, and in ER by 12 (63.2). Protocols for pain treatment at triage and/or ER were available in less than half of the hospitals. All protocols used at triage concerned pharmacological pain management, while only 3 included also non-pharmacological procedures. Only 2 EDs reported use of EMLA in over 50% of cases.

**Table 2:** Policies and reported practices towards pain management in children

	**At triage**	**In ER**
	**n (%)**	**n (%)**
**Pain assessment:**		
Never	**0** (0.0)	**1** (5.3)
Sometimes	**10** (52.6)	**10** (52.6)
Always	**9** (47.4)	**8** (42.1)
**Use of algometric scales:**		
No	**6** (31.6)	**10** (52.6)
Yes	**13** (68.4)	**9** (47.4)
**Results of pain assessment are recorded:**		
No	**5** (26.3)	**7** (36.8)
Yes	**14** (73.7)	**12** (63.2)
**Availability of protocols for pain treatment:**		
No	**11** (57.9)	**10** (52.6)
Yes	**8** (42.1)	**9** (47.4)
**Pain level contributes to priority determination at triage:**		
No	**11** (57.9)	
Yes	**8** (42.1)	
**Use of EMLA cream if blood sampling is anticipated:**		
Never/almost never (<10% of cases)	**12** (63.1)	
Sometimes (10-50% of cases)	**5** (26.3)	
Often/Always (>50% of cases)	**2** (10.6)	
Analgesic drugs mentioned in protocols (when available):*		
Non steroidal anti-inflammatory drugs (NSAIDS)		**10** (52.6)
Paracetamol		**10** (52.6)
Opioids		**6** (31.6)
Adjuvants		**5** (26.3)
**Pain reassessment:**		
Never/almost never (<10% of cases)		**2** (10.5)
Sometimes (10-50% of cases)		**6** (31.6)
Often (51-90% of cases)		**8** (42.1)
Always/almost always (>90% of cases)		**3** (15.8)
Parental role in the emergency room:*		
Entering with the child		**19** (100)
Being present during painful procedures		**17** (89.5)
Holding the child during blood sampling		**11** (57.9)

*Answers were not mutually exclusively.

For ER only, the type of analgesic drugs recommended in the protocols was investigated (Table 2). Non steroidal anti-inflammatory drugs (NSAIDs) and paracetamol were recommended with equal frequency (52.6%), while oppioids and adjuvants were recommended by 31.6 and 26.3% of departments respectively. Pain reassessment after treatment was carried out in over half of the cases by 11 hospitals (57.9%).

All hospitals allowed parents’ presence in the ER, and most (17, 89.9%) allowed them to stay also when painful procedures were carried out. Eleven hospitals (57.9%) allowed parents to hold their child during blood sampling.

**Figure 1:**
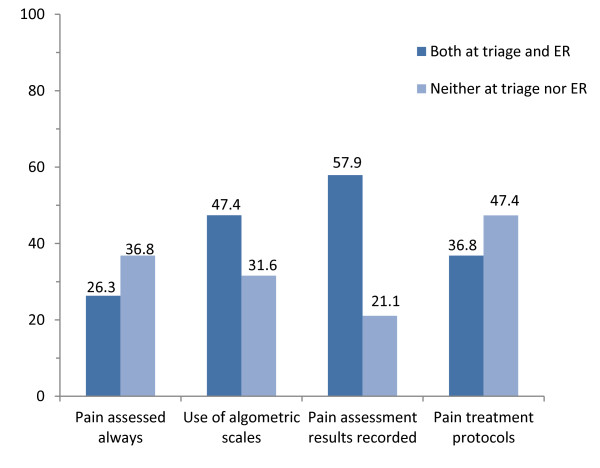
Proportion (%) of hospitals implementing appropriate pain management polices both and neither at triage and in the emergency room (ER).

To account for the paired nature of results collected at triage and in ER, we computed the proportions of hospital reporting appropriate policies both and neither at triage and in ER (Figure 1). The highest proportion of appropriateness both at triage and ER concerned recording of pain assessment results (57.9% of hospitals), followed by use of algometric scales (47.4%), presence of protocols (36.8%), and by routine (i.e. “always”) pain assessment (26.3%).

**Figure 2:**
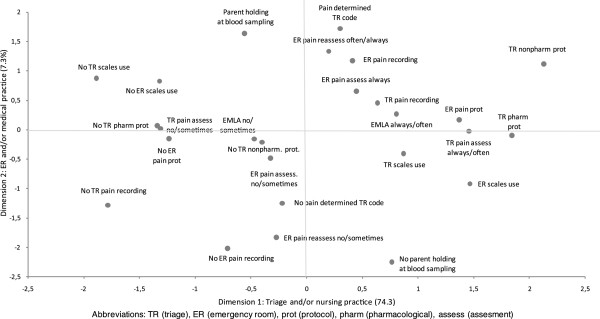
Results of multiple correspondence analysis: pain management map of the 19 Italian emergency departments.

When the variables related to pain management at triage and in the ER were considered together in a multiple correspondence model, two main dimensions emerged (Figure 2). The first, represented by the horizontal axis, explained the largest part of the variance (74.3%), and mostly included the pain management policies at triage: pain assessment and recording at triage and application of EMLA cream before venipunture. Use of algometric scales and availability of pain treatment protocols both at triage and in the ER also contributed to this dimension. In contrast the second dimension, on the vertical axis (7.3% of the variance only), appeared to include mainly pain policies in the ER: pain assessment in the ER and recording of results, pain reassessment after treatment, and child holding by parents at blood sampling. Also the use of pain as criterion for priority coding at triage contributed to this dimension, possibly because such a rule is unlikely to be determined by nurses only.

**Figure 3:**
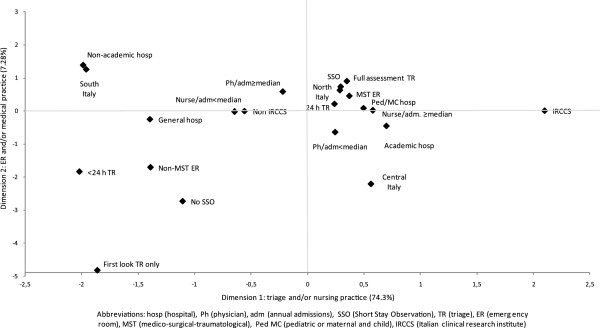
Results of multiple correspondence analysis: plot of hospital characteristics predicting pain management policies.

When the hospital and ED characteristics were plotted over the graph (Figure 3), information about their association with pain management policies emerged. Pediatric and maternal and child hospitals, those located in the North of Italy, equipped with medico-surgical-traumatological (MST) ED, full assessment triage over the 24 hours, and SSO were placed in the upper right quadrant of the map, corresponding to the positive sides of both axes and linked to the most appropriate pain management practices both at triage and in ER. Being a clinical research institute and having a nurses to admissions ratio ≥ median value were also close to this cluster, but their positive influence concerned specifically the horizontal axis, indicating appropriate pain management at triage. General hospitals, those with a non-MST ER, no SSO, triage < 24 hours and carried out as first look only were associated with less appropriate pain management reported practices. Academic hospitals and those in central Italy scored positively for triage, but not for ER practices. The opposite was true for non academic hospitals and for those located in the South of the country.

## Discussion

This study shows that policies and practices towards children’s pain management in Italian pediatric emergency departments are still sub-optimal. Pain assessment both at triage and in the ER is performed “always” by 26% of surveyed hospitals only. About one third do not use algometric scales, and 21% do not document the results of pain assessment on clinical records. Almost half of the EDs (47.4%) do not have local protocols for pain treatment. Only 3 hospitals routinely reassess pain after treatment, and only 2 use EMLA cream when venipuncture is anticipated.

We found that hospitals in the North of Italy, those equipped with a medico-surgical and traumatological ED and with SSO, and those providing full assessment triage over the 24 hours are more likely to report appropriate policies for pain management both at triage and in the ER. Pediatric and maternal and child hospitals also belong to this group, and this finding is in agreement with previous studies indicating better pain management in pediatric than in general hospitals [7,15].

In Italy, pain management at triage is mainly carried out by nurses, while practices in the ER are more dependent on the physicians’ attitudes and needs. Our analysis showed that a nurse to admissions ratio larger than the median is associated with better pain management at triage. Southern hospitals, that show poorer pain management practices at triage, also have the lowest nurses to admissions ratio. On the other hand, a less favourable physicians to admissions ratio (≤ median values) appears associated with poorer practices in the ER, confirming the relevance of staff resources and patient crowding to adequate pain management [22,23].

We found that local protocols for pain management, when available, mostly recommended the use of paracetamol and NSAIDS, while opioids were mentioned by six EDs only. Fear of adverse side effects and potential for addiction following use of opioids in children are common [4] and not completely ill-founded [24]. However, in-hospital opioid analgesia for children and even neonates is generally recommended in case of moderate to severe pain [1,4,25]. Measures such as vital signs monitoring and availability of naloxone are important to guarantee safety, as well as specific education of physicians [1].

A positive finding of our study is represented by the reported policies towards parents, with all surveyed hospitals allowing parents to stay in the ER with their child, even during painful procedures. Previous studies carried out in Italy in settings different from emergency care documented more restrictive policies towards parents. Repeated comparisons between several European countries showed that Neonatal Intensive Care Units in Italy were less likely to allow free parental visiting and participation in the care of the babies [26-29], and less babies’ holding and kangaroo care [30]. Similar restrictive policies were documented in a survey of Pediatric Intensive Care Units [31]. It is possible that the health personnel is more reluctant to allow routine parental presence in the highly protected environment of intensive care than in emergency rooms. Alternatively the present, more recent study may suggest increasing acceptance of the principles of family centered care in Italy.

Only 58% of hospitals, however, allowed parents to hold their child during blood sampling, and only 2 reported the use of local anaesthesia with EMLA. Venipuncture for blood sampling or to start intravenous line is a very common and simple procedure in emergency care. In children however it may be associated with greater technical difficulties and considerable patient fear and anxiety, which may result in distress to the parents and to the staff performing the procedure. The impact of unmanaged needle insertion pain may be long lasting [32]. Several studies have shown that in this setting the presence of parents may be an asset, provided adequate preparation and the offer of an active role, such as coaching their child to cope, distraction, and even assistance with patient positioning [33]. Venipuncture with the child held by a parent in upright position has been shown to reduce patient distress and increase parental satisfaction, without negatively influencing the performance of the procedure [34,35].

This study has limitations. While the sampling of pediatric and maternal and child hospitals was exhaustive, providing a reliable picture of their policies, only a convenience sample of general hospitals with separate pediatric triage was recruited. Our survey focussed on organization of EDs and policies, while actual care of individual children was not investigated. A second part of this project, however, will investigate in the same sample of hospitals pain management at the patient level, using pediatric admissions for headache as indicator. This will allow us to link the EDs policies and organisation to pain assessment and treatment in the individual patient, and explore their relationship. Previous studies have shown that actual practices tend to be, if anything, poorer than reported [5].

Medical progress in recent decades has allowed increasingly better methods of pain management. Validated instruments to assess pain at different pediatric ages have been developed [3]. Pharmacological advances have enhanced the possibilities for effective and safe pain prevention and treatment, and guidelines specific for the emergency care setting are now available [25]. Yet, as this study shows, local policies and practices are constantly lagging behind results of scientific research and development of guidelines.

Implementing a pain-free clinical practice in the ED requires overcoming both cultural and organizational barriers as well as persistent myths about the pediatric pain experience [2]. A multidisciplinary paradigm shift is needed, placing highest priority on the prevention, assessment and treatment of pain in children. Interventions such as development of local policies, staff educational programs, and nurse-initiated pain treatment should be applied together as an integrated strategy, and periodically reinforced [36]. A partnership should be formed with parents, who need to be informed about the dangers of pediatric pain, educated about the importance of pain prevention and management, involved in pain assessment and given an active role in non-pharmacological treatment [37]. The evaluation of existing hospital policies and practices is only a first step towards these goals, but may provide the impetus to start change.

## Conclusions

Despite availability of national and international guidelines, pediatric pain management is still sub-optimal in Italian emergency departments as regards presence of local protocols, use of algometric scales, pain reassessment after treatment, and use of EMLA for venipuncture. As appropriate pain management is recognized as an important component of pediatric care, strategies including development of local policies, staff educational programs, and parental involvement in pain assessment should be implemented.

## Abbreviations

ED: Emergency department; ER: Emergency room; IRCCS: Istituto di ricovero e cura a carattere scientifico (Clinical Research hospital); MCA: Multiple correspondence analysis; MST: Medico-surgical traumatological; NR: Nurses to admissions ratio; NSAIDS: Non steroidal anti-inflammatory drugs; PR: Physicians to admissions ratio; SSO: Short stay observation.

## Competing interests

The authors declare that they have no competing interests.

## Authors’ contributions

PF carried out multivariable analysis and prepared the first draft of the manuscript. FB initiated the study, coordinated the PIPER Study Group, drafted the questionnaire and participated in the interpretation of results and in the critical revision of the manuscript. VL and SP coordinated data collection and management, and contributed to data analysis. MC designed the study, participated in the preparation of the questionnaire, supervised statistical analyses, and contributed to the drafting of the manuscript. TZ, CT, GM and NP participated in the preparation of the questionnaire, contributed to the interpretation of results, and critically revised the manuscript. All authors read and approved the final manuscript.

## Pre-publication history

The pre-publication history for this paper can be accessed here:

http://www.biomedcentral.com/1471-2431/13/139/prepub
